# Developmental Cycle and Genome Analysis of *Protochlamydia massiliensis* sp. nov. a New Species in the *Parachlamydiacae* Family

**DOI:** 10.3389/fcimb.2017.00385

**Published:** 2017-08-31

**Authors:** Samia Benamar, Jacques Y. Bou Khalil, Caroline Blanc-Tailleur, Melhem Bilen, Lina Barrassi, Bernard La Scola

**Affiliations:** Unite de Recherche sur les Maladies Infectieuses et Tropicales Emergentes, UM63 Centre National de la Recherche Scientifique 7278 IRD 198 Institut National de la Santé et de la Recherche Médicale U1095, Institut Hospitalo-Universitaire Mediterranee Infection Marseille, France

**Keywords:** *Chlamydiae*, *Vermamoeba vermiformis*, co-culture, CRISPR, *Protochlamydia massiliensis*

## Abstract

Amoeba-associated microorganisms (AAMs) are frequently isolated from water networks. In this paper, we report the isolation and characterization of *Protochlamydia massiliensis*, an obligate intracellular Gram-negative bacterium belonging to the *Parachlamydiaceae* family in the *Chlamydiales* order, from a cooling water tower. This bacterium was isolated on *Vermamoeba vermiformis*. It has a multiple range of hosts among amoeba and is characterized by a typical replication cycle of *Chlamydiae* with a particularity, recently shown in some chlamydia, which is the absence of inclusion vacuoles in the *V. vermiformis* host, adding by this a new member of Chlamydiae undergoing developmental cycle changes in the newly adapted host *V. vermiformis*. Draft genome sequencing revealed a chromosome of 2.86 Mb consisting of four contigs and a plasmid of 92 Kb.

## Introduction

The *Chlamydiae* is an assemblage of obligate intracellular bacteria that was recently re-classified into the *Chlamydiales* order. These microorganisms are unified by their unique biphasic developmental cycle (Horn, 2008), dependency on eukaryotic cells, and their pathogenicity toward animals and humans. In the host cell, these bacteria can enter an endosymbiotic stage, replicate, and spread after cell lysis (Longbottom and Coulter, 2003). Lately, some developmental cycle variations regarding the presence or absence of chlamydial inclusion vacuoles, have been reported and linked to the host, significantly adding to our knowledge of this field of chlamydia (Bou Khalil et al., 2017). Moreover, genetic exchange can occur between the *Chlamydiae* and its associated host (Gimenez et al., 2011) through a widespread system that can be described in two major groups: those that facilitate DNA transfer, such as the F-like system, and those that help in protein and nucleoprotein translocation, such as P- and I- like systems.

Until the 1990s, studies focused on chlamydia as a member of *Chlamydiae*. It was thought to be the single family of the *Chlamydiales* order (Kahane et al., 1993), which includes 11 species. Although our understanding of the significance and diversity of bacteria belonging to the family *Chlamydiaceae* has been well-established due to intensive research over the last 50 years, recent studies have also revealed that this family only represents the “leading edge” in terms of its diversity, especially given the recent discovery of eight novel genetically related families. At the genomic level, *Chlamydiae* is known to have been evolutionary disconnected from other bacteria almost a billion years ago (Everett et al., 1999; Greub and Raoult, 2003). These bacteria can be identified by the presence of conserved indels and by several signature proteins that are only present in several *Chlamydiae* species (Horn et al., 2004; Griffiths et al., 2005). The exploration of the richness of this phylum is of interest in terms of deciphering hidden pathogenicity and new features in this vast world of bacteria. In this paper, we report the isolation of a new *Chlamydiae* species, *Protochlamydia massiliensis* sp. nov., belonging to the *Protochlamydia* genus. We describe its genome features and its developmental cycle.

## Materials and methods

### Isolation, production, developmental cycle, and quantification procedures

*P. massiliensis* was isolated from a cooling tower in the Vaucluse region of France, as previously described (La Scola et al., 2000; Pagnier et al., 2015), using *Vermamoeba vermiformis* as a cell support for amoeba co-culture. For production, after being rinsed with PAS (Page's amoeba saline) and suspended in a starvation medium (Bou Khalil et al., 2016) with a final concentration of 10^6^ amoebas/ml, *V. vermiformis* was infected with a *P. massiliensis* suspension at a multiplicity of infection (MOI) of 10, in two 75-cm^2^ culture flasks at 30°C. Washing with PAS buffer was performed after 1 h (H0) of incubation to eliminate non-internalized bacteria. Next, 10 ml of infected cultures was incubated at 30°C in new culture flasks alongside a negative control consisting of a flask containing only amoeba for further analysis.

Of these 10 ml, 500 μl of culture suspension was used for the preparation of five slides through cyto-centrifugation in order to perform Gram staining, Gimenez staining and DAPI nucleic acid labeling (Molecular probes, Life Technologies USA) at the following stages respectively: H0, H2, H4, H6, H8, H12, H18, H24, H30, H36, H42, and H48. In addition, 500 μl of the prepared culture suspension was used for DNA extraction and molecular analysis. In short, bacterial growth and count were calculated using real time PCR assays, in order to assess the correlation between bacterial concentration and cycle threshold (Ct.), 200 μl of each co-culture at every infection time point of the cycle (H0–H48) were used for DNA extraction by EZ1 DNA Tissue Kit (Qiagen, Hilden, Germany) according to the manufacturer's instructions on a CFX96TM thermocycler (BioRad Laboratories Inc.). The real-time PCR primers used were the following: Forward: 5′GCTCCGATTCAGCGAATACT 3′, Reverse: 5′GTCTGCTCTTCCATTCCCATAA3′, and Probe: 5′CTGCTAAGCATGTTGCTAAGCTTGGC 3′. Amoebal quantification was performed on counting slides (kovaslides, HYCOR biomedical Inc., 90 California, USA).

The remaining 9 ml was used for transmission electron microscopy after pellet fixation. For transmission electron microscopy processing, all specimen fixation, embedding, cutting, observation, and analysis procedures were performed as previously described by Bou Khalil et al. (2016). For the host range, the supernatant of a 1-week-old culture flask of *V. vermifromis* infected with *Chlamydiae* was collected and filtered through a 5 μm pore-size filter and washed three times with modified PAS. The later was used for inoculation onto *A. castellanii* (strain Neff 30010) and *A. polyphaga* (strain Linc-AP1) at an MOI of 10 in a 24-well microplate seeded with 10^6^ *Amoebae* and containing 1 ml of PAS. The microplates were then incubated at 32°C (*A. castellanii*) for 5 days following centrifugation at 1,500 × g for 30 min. Amoebas were observed daily for lysis and 500 μl from the co-cultures was taken daily for DNA extraction and assessment of the number of chlamydial DNA copies using real-time quantitative PCR.

Quantitative replication values were statistically compared using the Mann whitney test and a *p*-value < 0.05 was considered to reflect a significant difference.

### Sequencing, assembling, and genome annotation of *protochlamydia massiliensis*

The genomic DNA of *P. massiliensis* was sequenced using a MiSeq Technology sequencer (Illumina, Inc., San Diego, CA). Four Illumina short-insert paired-end libraries were performed with an average insert size of 251 bps along with an Illumina long-insert paired-end library with an insert size of 520. Libraries and sequencing were performed as previously described (Lo et al., 2015). Reads were then assembled using the *de novo* method and the SPAdes-3.0.0 software (Bankevich et al., 2012). The non-coding genes and miscellaneous features were generated using RNAmmer (Lagesen et al., 2006), ARAGORN (Laslett and Canback, 2003), Rfam (Griffiths-Jones et al., 2003), and Infernal (Nawrocki et al., 2009). Coding DNA Sequences (CDSs) were predicted using Prodigal (Hyatt et al., 2010) and functional annotation was achieved using BLAST+ (Camacho et al., 2008) and HMMER3 (Eddy, 2011) against the UniProtKB database. Data for *P. massiliensis* were submitted to the EMBL database and were assigned Bio-projects number PRJEB6590; the accession numbers for the genome in the EMBL database are CCJF01000001-CCJF01000005.

### Phylogenetic tree construction

Phylogenetic analyses were performed for the *Chlamydiae* genes and the corresponding gene sequences available on the NCBI database. Multiple sequence alignments were performed using MUSCLE (Edgar, 2003) and curated using Gblocks (Talavera and Castresana, 2007). Phylogenetic trees were generated using the PhyML Maximum Likelihood algorithm and visualized using MEGA v5 (Tamura et al., 2011).

## Results

### Culture and developmental characteristics

**Figure 1:**
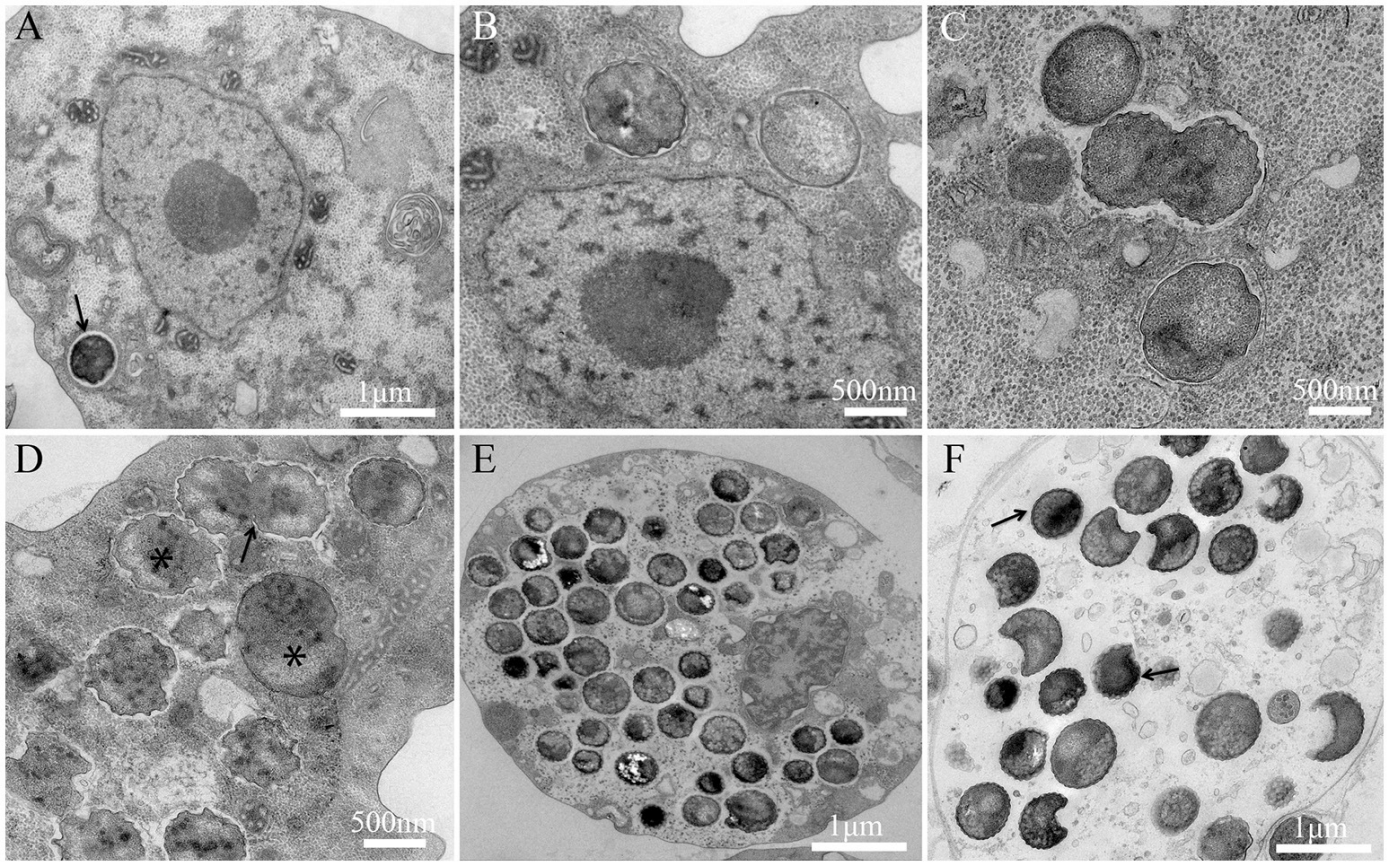
Developmental cycle of *P. massiliensis* in *V. vermiformis*. **(A)** Internalization of a phagocytized *P. massiliensis* elementary body (EB), by a trophozoite of *V. vermiformis* at H0 p.i. **(B)** Ultrathin section of an amoeba harboring reticulate bodies (RBs) near the nucleus undergoing the replicative stage, where an increase in size and a decrease in density can be observed. **(C)**, **(D)** Replicative stage at H12, H16, H18 p.i showing an increased number of *P. massiliensis* particles and hypodense RBs at different stages of morphogenesis (asterisks in **D** marking RBs. Some bacteria at the typical binary fission stage are also observed (black arrow in **D** showing typical binary division, and the constriction of some RBs can be observed). **(E)**, **(F)** End of the replicative stage where, after growth and binary division, the RBs start reorganizing and condensing to form infectious EBs showed by black arrows. Different stages of the *P. massiliensis* developmental cycle can be detected, and RBs and EBs can be observed simultaneously, scattered inside in the host cytoplasm.

The bacterium is strictly intracellular and unable to grow outside amoeba. To date, all attempts to grow this bacterium on agar media and nutritive broths (PYG, Trypticase soy broth) under several axenic conditions (aerobic, anaerobic, and microaerophilic atmospheres) have failed. For the replicative cycle, bacterial embedded bodies (EBs) are phagocytized and internalized in a vacuole inside the amoebal cytoplasm and can be seen near the nucleus (Figure 1A). At H4, H6, H8 post-infection, bacteria begin to lose their electron-density, progressively increase in size, and form reticulate bodies (RBs) (Figures 1B,C). Between H8 and H12, bacterial bodies continue to grow and form large accumulations of material, and binary division of the bacteria can be observed (Figures 1C,D). Between H24 and H36, the division continues to increase and the bacteria occupy the entire amoeba cytoplasm (Figures 1E,F). After a period of growth and division of a typical asynchronized cycle of *Chlamydiae*, the RBs reorganize between, approximately, H36 and H42 p.i., condensing to form infectious and pre-mature EBs (Figure 1E) that mature into highly condensed ones, leading to cell burst (Figure 1F). The *P. massiliensis* grew in *V. vermiformis, A. polyphaga* and *A. castellanii*, and a cytopathic effect was seen 48 h after infection. Complete lysis was observed within 96 h. In contrast, the non-infected cultures of each host cell, used as negative controls, showed no detectable loss of amoebae over the same experimental timeframe.

**Figure 2:**
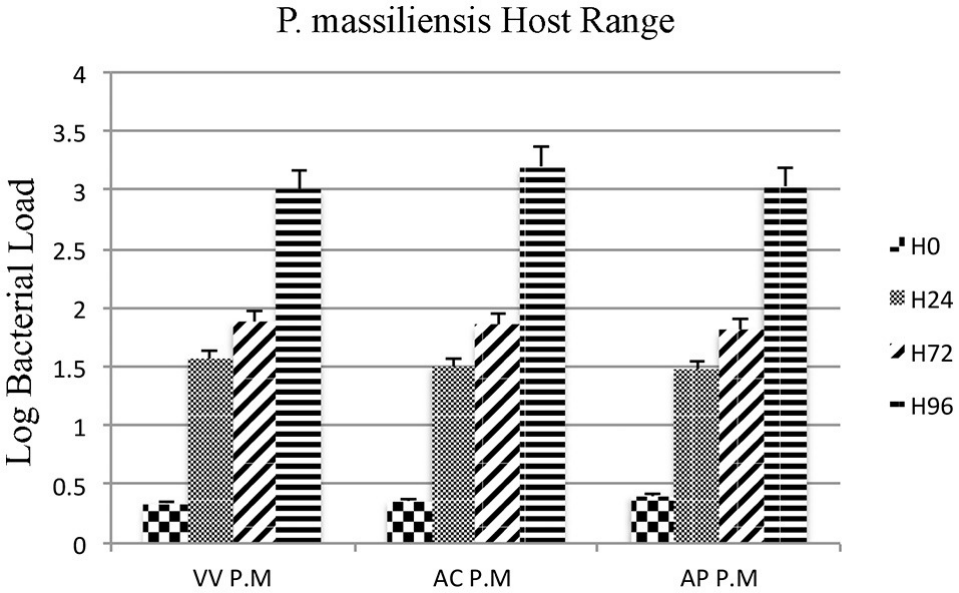
Host range of *P. massiliensis*. Histogram of *P. massiliensis* growth in three types of amoeba; *V. vermiformis, A. castellanii*, and *A. polyphaga*, measured by real-time PCR for 4 days post-infection. Data are the mean *SD* from three independent experiments performed in triplicate. (H0, H24, H72, and H96 correspond to different time points in hours). (VV P.M = *V. vermiformis* infected with *P. massiliensis*, AC P.M = *A. Castellanii* infected with *P. massiliensis*, AP P.M = *A. polyphaga* infected with *P. massiliensis*). The Y-axis corresponds to the log of bacterial load (the log values are obtained after conversion of the Cycle threshold (Ct.) values based on standard curves performed with serial 1:10 dilution starting with 10^7^ bacterial particles). This relative quantification by real-time PCR shows the increase in bacterial multiplication in all tested hosts cells. Statistical test showed a *p*-value > 0.05.

**Figure 3:**
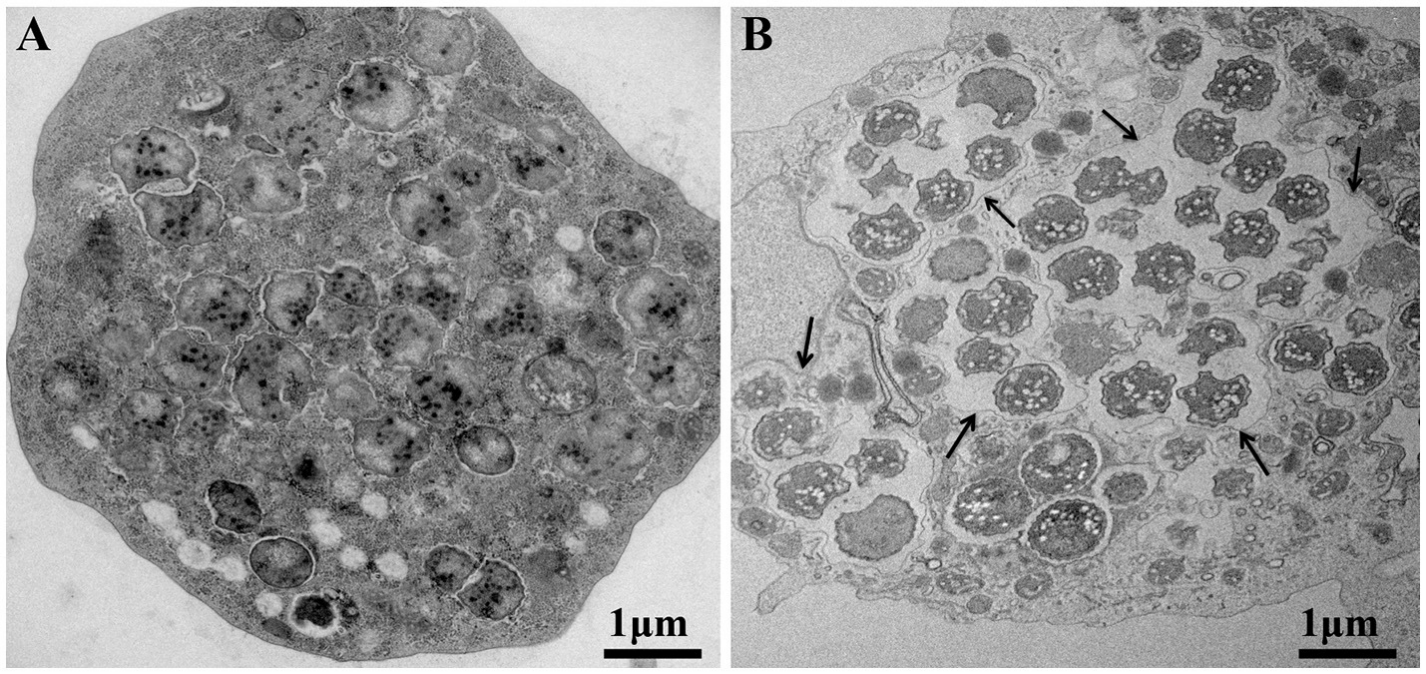
The *P. massiliensis* developmental cycle with typical inclusion vacuoles in *A. castellanii*. Both host cells were studied at the same time points of infection. **(A)** *P*. *massiliensis* infecting *V. vermiformis*, where no inclusion vacuoles can be seen in the infected amoeba. Bacteria are detected in the cytoplasm. **(B)** *P*. *massiliensis* infecting *A. castellanii and* inclusion vacuoles surrounding the bacteria in the cytoplasm are clearly observed (black arrows).

Moreover, the culture and relative quantification by real-time PCR showed an increase in bacterial multiplication in all three amoebae strains at H24, but particularly at H72 and H96, with an approximate three-log increase in the number of bacteria per milliliter in 4 days (Figure 2). No significant statistical difference was found in the quantitative replication values between the three tested hosts, where the realized Mann whitney test showed a *p*-value > 0.05. When comparing the replicative cycle of *P. massiliensis* in *A. castellanii* and in *V. vermiformis* at the same time points of infection, no inclusion vacuoles could be detected in the case of *V. vermiformis* and bacteria were directly identified in the cytoplasm (Figure 3A). However, we clearly observed the presence of inclusion vacuoles filled with *P. massiliensis* in the case of *A. castellanii*, where bacteria appear to grow in vacuoles in the cytoplasm, forming morula-like shapes (Figure 3B).

### Taxonomy and general features

**Figure 4:**
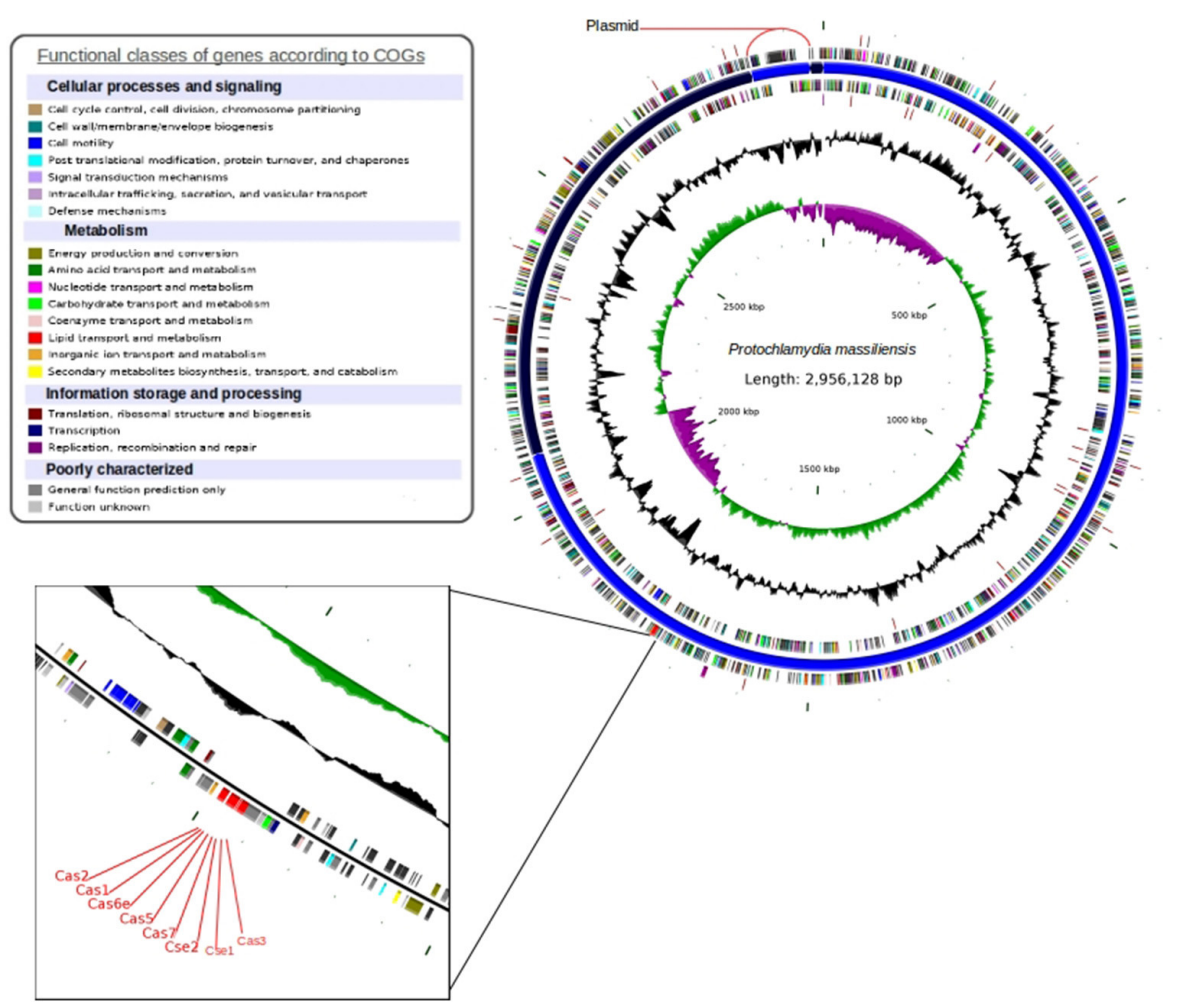
Circular representation of the *P. massiliensis* genome. Circles from the center to the outside: GC skew (green/purple); GC content (black); tRNA (dark red); rRNA (purple); tmRNA on the forward strand; genes on the forward strand colored by COGs categories; scaffolds in alternating blue; genes on the reverse strand colored by COGs; tRNA (dark red); and rRNA (purple) on the reverse strand.

The *P. massiliensis* genome consists of a chromosome sequence assembled into four contigs (Figure 4). This sequence was estimated to have 2,864,073 pbs and a GC content of 42.8%. In total, 2,389 Protein Coding Sequences (CDS) were identified as well as six sets of rRNA (two genes are 5S rRNA, two genes are 16S rRNA, two genes are 23S rRNA) and 39 tRNA genes. A total of 1,204 genes (50.39%) were attributed a putative function (by cogs or by NR blast). In addition, 589 genes were identified as ORFans (24.65%), and the remaining genes were annotated as hypothetical proteins (21.05%). The fifth contig is 92,055 bps with a GC content of 40.87%, represented a circular plasmid. The average read depth of this plasmid (294x) compared to the chromosome (153x) suggests that the plasmids are present in double copy per cell. After annotation, hypothetical functions can be assigned to more than 80% of the plasmid genes.

The phylogenetic tree of the 16S rRNA, built with a representative set of chlamydia sequences available in the NR database, clusters *P. massiliensis* within the *Protochlamydia* genus (Figure S1). The bootstrap relating *P. massiliensis* to the other members of the *Protochlamydia* genus was 100%. The chlamydial taxonomy based on the 16S rRNA sequence similarity and phenotypic characteristics was used for chlamydial description. However, the *Chlamydiales* order exhibits few phenotypic differences. Moreover, Tamisier et al. showed that many of the current bacterial species with validly published names do not respect the 95 and 98.7% thresholds usually considered as delineating genus and species respectively (Rossi-Tamisier et al., 2015). Pillonel et al. recommended using the 16S and 23S genes combined with 9 protein sequences to precisely classify newly discovered isolates at the family, genus, and species levels (Pillonel et al., 2015). We used Pillonel's classification method, which showed that *P. massiliensis* is a new *Protochlamydia* species.

### CRISPR sequence analysis

A CRISPR subtype I-E locus was identified in *P. massiliensis* (Bertelli et al., 2016). In a phylogenetic tree built using 43 cas1 proteins (Figure S2), *P. massiliensis* groups within the *Proteobacteria* cluster with a higher bootstrap value (100%). A total of 15 spacers were identified in the *P. massiliensis* genome (Figure S3), showing 40.80% GC content, and 42.75% genomic GC content. The *P. massiliensis* spacers have no significant identity in the NR database. However, a search against the “My CRISPRs DB” database (Grissa et al., 2007) enabled us to correlate a part of its sequence to a spacer from *Spirochaeta thermophila* CRISPR. In spite *P. massiliensis* was isolated from warm water of cooling tower, there is no evidence now of relationships between this later and *S. thermophila*, a strict anaerobic extracellular extremely thermophilic spirochete isolated from salt water hot springs. *P. massiliensis* CRISPR repeats have 28 bp length, the first fourteen repeats sequences displayed total identity and four substitutions were observed in the last two repeat sequences (C/T, A/G, and A/T).

## Discussion

The warm water from cooling towers represents a suitable environment for microorganism proliferation and biofilm formation. In this study, we report the isolation and description of a novel chlamydia from a cooling tower, which will help us better, understand chlamydial evolution and obligate intracellular parasitism. Similar to *Neochlamydia hartmanellae*, an endoparasite of *V. vermiformis* (Horn et al., 2000), *Rubidus massiliensis* (Bou Khalil et al., 2016), and *P. phocaeensis* (Bou Khalil et al., 2017), *P. massiliensis* was able to grow without any inclusion vacuoles in *V. vermiformis* and showed inclusion vacuoles in *A. castellanii*, with an interesting point, which is having the same growth level in both hosts. Investigations of these findings are ongoing to improve understanding. As we noticed and cited in our previous works on amoeba associated Chlamydiae and the inclusion vacuoles process with many hypothesis (Bou Khalil et al., 2017), which made us speculate that this type of *Chlamydiae* developmental cycle is probably due to its host. In fact and for more clarification, replicative vacuoles process requires a complex interplay of host pathways and bacterial factors. As it is the case for *P. phocaeensis* (Bou Khalil et al., 2017), and *P. massiliensis*, the escape from the inclusion vacuoles may be due either to atypical host pathways or to the composition of Chlamydiae proteins. The fact that these chlamydia were able to infect other amoebae and to form inclusion vacuoles leads us to believe that the break in the inclusion vacuole is induced by the *V. vermiformis*′ protein. The inclusion vacuole provides the pathogen with a complex set of interactions between chlamydial inclusion and host-cell trafficking pathways, facilitating the acquisition of essential host-derived nutrients. The inability of chlamydia to form an inclusion vacuole in *V. vermiformis* orientate us to more investigate the difference between the amoeba hosts and correlate this issue with our findings to better understand this process. For this, we are focusing on the hosts, as we are still investigating this issue, with more techniques at different levels going from the biology or morphological and physiological differences between the hosts to the comparison of their genomes when available, in order to correlate all potential findings with the cluster of all available Chlamydiae capable of infecting these same hosts.

Analysis of the complete genome sequence of *P. massiliensis* enabled us to detect a chromosome of 2.85 Mb and a plasmid of 92 Kb. The Pillonel et al. taxonomy method (Pillonel et al., 2015) allowed us to classify *P. massiliensis* as a new *Protochlamydia* species. The CRISPR loci identified in the genome of *P. massiliensis* had 40.80% GC content, while the genomic GC content is 42.75%. This may indicate that the CRISPR was laterally transferred from a low-GC bacterium. Nevertheless, Cas1 are present in all CRISPRs, and are used for CRISPR classification and evolution analysis. A phylogenetic tree using 43 cas1 proteins revealed the clustering of *P. massiliensis* cas1 with *Proteobacteria* Cas1 proteins. This implies that these bacteria may share the same CRISPR Loci origin. Although CRISPRs are known to have conserved repeat sequences, some single nucleotide polymorphisms, usually at its 3' end, may be observed (Horvath et al., 2008). In the case of *P. massiliensis*, CRISPRs showed four substitutions in its two last repeats. Moreover, *P. massiliensis* harbored 15 spacers with 33 bps. Spacers are known to be flanked by two consecutive CRISPR repeats and to play a role in conjugation with Cas proteins in surveillance and adaptive immune systems. These short sequences are derived from the infecting “pathogen” (Barrangou et al., 2007; Deveau et al., 2008; Horvath et al., 2008) (phage, plasmid determinants, viruses), and their presence in the CRISPR sequence confers the bacterium with an acquired “immunity” system against “pathogens” which contains an identical proto-space. Blasting *P. massiliensis* spacers against the NR database yielded no significant match. However, blasting against the “My CRISPRs DB” (Grissa et al., 2007) enabled a partial identity with a spacer from *S. thermophila* DSM 6578 CRISP to be identified. Therefore, we suggest that these two bacteria may have “immunity” against the same “pathogen.”

### Description of “*Protochlamydia massiliensis* sp. nov.”

(mas.si.li.en′sis, L. fem. adj. *massiliensis*, referring to Massilia, the Latin name for Marseille, where the strain was characterized). Phylogenetic position, *Chlamydiales* order; Gram-negative; mature infectious particles have coccus-shaped morphology 0.6 ± 0.2 mm in size; the access number for the genome at Gen Bank is NZ_CCJF01000000; this strain has been deposited in the CSUR (http://www.mediterranee-infection.com/article.php?laref=14&amp;titre=collection-de-souches Collection de Souches de l′Unite des Rickettsies) under reference CSUR (P2508). Does not grow on cell-free media; obligate intracellular pathogen of multiple amoebal hosts such as *A. castellanii, A. polyphaga*, and *V. vermiformis*. *P. massiliensis* has a development cycle with two morphological stages, typical of chlamydia, and multiplies through binary fission but lacks inclusion vacuoles in the *V. vermiformis* host.

## Author contributions

SB and JB contributed equally to this work. SB performed bioinformatic analysis and wrote the paper, JB performed isolation, TEM, and wrote the paper, CB conducted genome sequencing, MB and LB performed culture, BL designed the study and corrected the manuscript.

### Conflict of interest statement

The authors declare that the research was conducted in the absence of any commercial or financial relationships that could be construed as a potential conflict of interest.

## Supplementary material

Figure S1 *Chlamydiales* members clustering according a phylogenetic tree analysis. Maximum-likelihood (PhyML) phylogenetic tree calculated with JTT+G substitution model, with the RNA 16S sequences of 33 *Chlamydiales* members. Bootstrap proportion values are indicated at the node.

Figure S2 Type I-E CRISPR Cas1 proteins clustering, according a phylogenetic tree analysis. Maximum-likelihood (PhyML) phylogenetic tree calculated with JTT+G substitution model with the Cas1 protein sequences of 43 bacterial members. Bootstrap proportion values are indicated at the node.

Figure S3 CRISPR locus and its associated genes. Cas genes are colored according the protein type. Direct repeats and spacer sequences are given in the panel.
