# Supplementary figures and images for: Developmental Cycle and Genome Analysis of Protochlamydia massiliensis sp. nov. a New Species in the Parachlamydiacae Family
Source: Front Cell Infect Microbiol. 2017 Aug 31;7:385. doi: 10.3389/fcimb.2017.00385 (PMC5583166; doi:10.3389/fcimb.2017.00385)

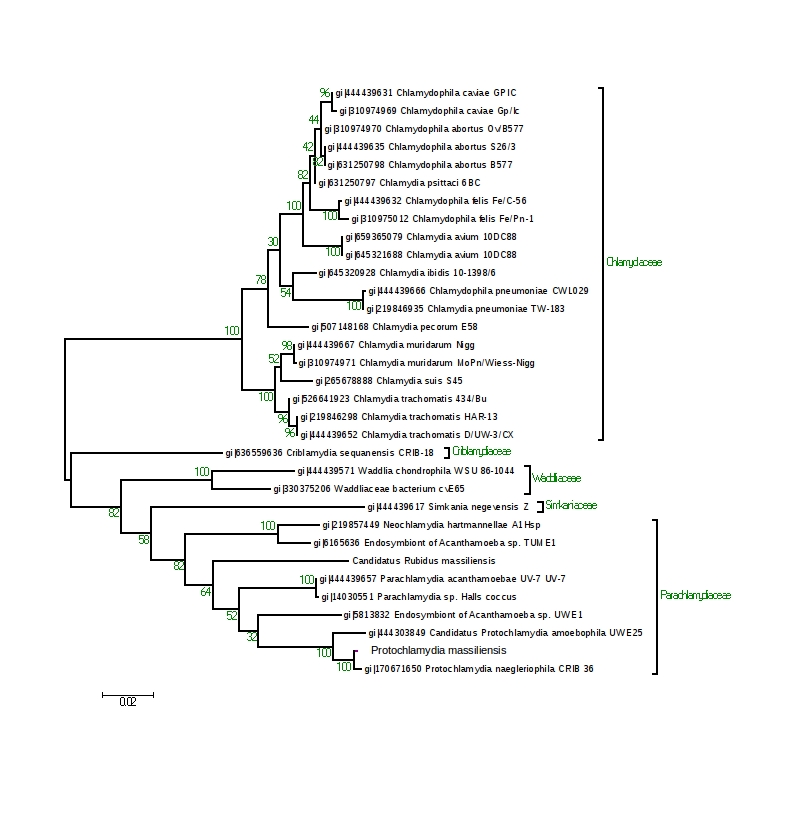

Supplement: Figure S1 — Chlamydiales members clustering according a phylogenetic tree analysis. Maximum-likelihood (PhyML) phylogenetic tree calculated with JTT+G substitution model, with the RNA 16S sequences of 33 Chlamydiales members. Bootstrap proportion values are indicated at the node. [file Image1.JPEG]

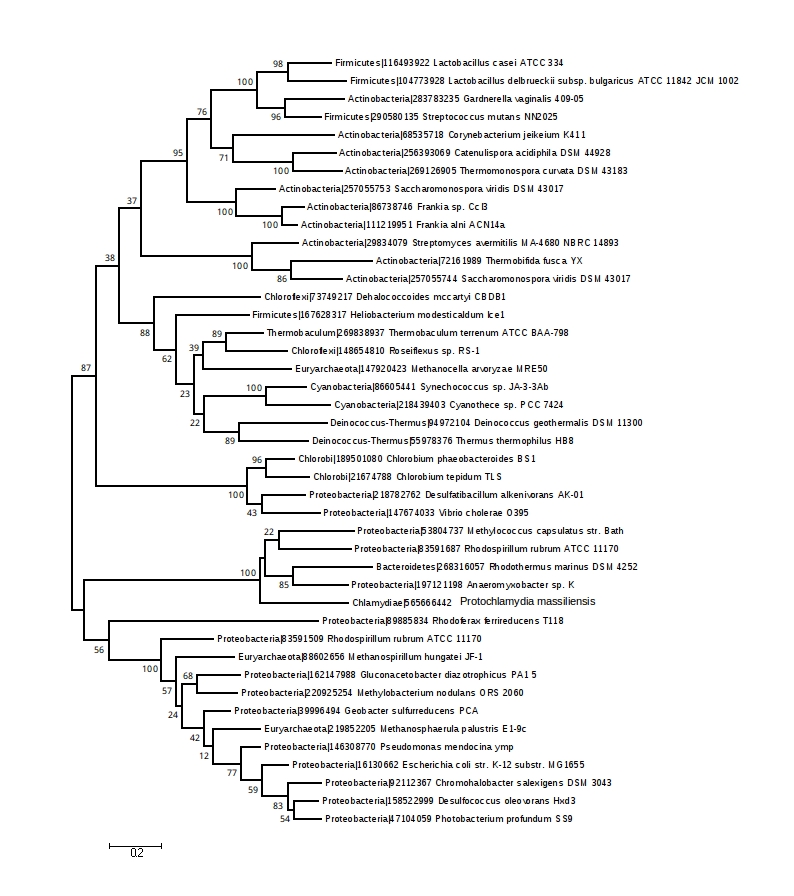

Supplement: Figure S2 — Type I-E CRISPR Cas1 proteins clustering, according a phylogenetic tree analysis. Maximum-likelihood (PhyML) phylogenetic tree calculated with JTT+G substitution model with the Cas1 protein sequences of 43 bacterial members. Bootstrap proportion values are indicated at the node. [file Image2.JPEG]

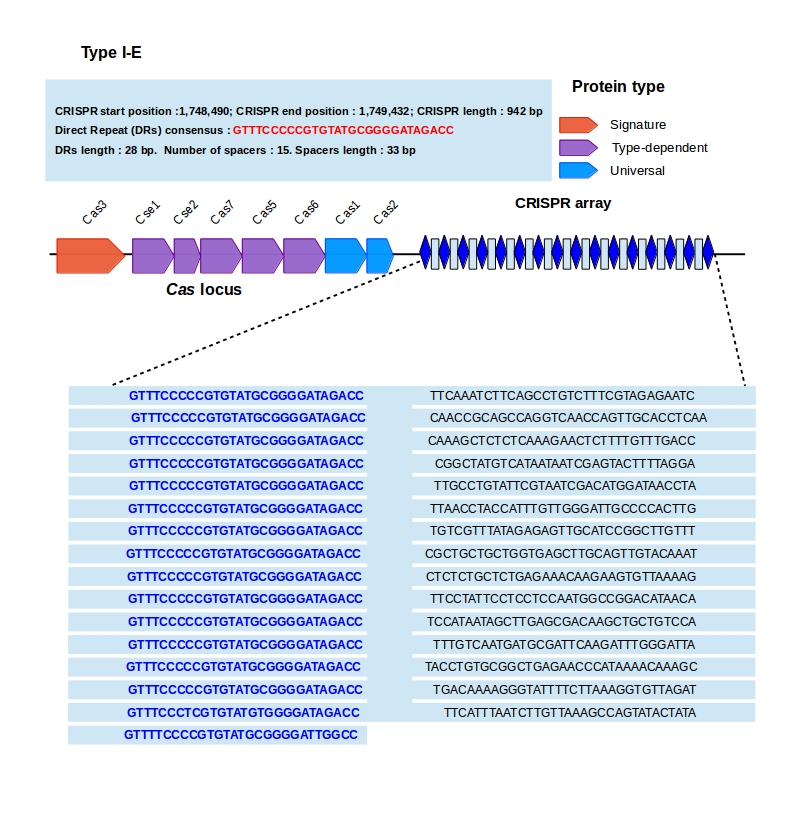

Supplement: Figure S3 — CRISPR locus and its associated genes. Cas genes are colored according the protein type. Direct repeats and spacer sequences are given in the panel. [file Image3.JPEG]
